# LncRNA MALAT1 shuttled by bone marrow-derived mesenchymal stem cells-secreted exosomes alleviates osteoporosis through mediating microRNA-34c/SATB2 axis

**DOI:** 10.18632/aging.102264

**Published:** 2019-10-26

**Authors:** Xucheng Yang, Junxiao Yang, Pengfei Lei, Ting Wen

**Affiliations:** 1 Department of Orthopedics, Xiangya Hospital, Central South University, Changsha 410008, P. R. China

**Keywords:** osteoporosis, bone marrow-derived mesenchymal stem cells, exosome, MALAT1, osteogenesis

## Abstract

Long non-coding RNAs (lncRNAs) have emerged as promising novel modulators during osteogenesis in mesenchymal stem cells (MSCs). Enhanced SATB2 has been demonstrated to promote osteogenic differentiation of bone marrow-derived mesenchymal stem cells (hBMSCs) in patients with osteonecrosis. Preliminary bioinformatic analysis identified putative binding sites between microRNA-34c (miR-34c) and metastasis-associated lung adenocarcinoma transcript 1 (MALAT1) or miR-34c and SATB2 3’UTR. Thus, the current study aimed to clarify the potential functional relevance of MALAT1-containing exosomes from BMSCs in osteoporosis. The extracted exosomes from primary BMSCs were co-cultured with human osteoblasts (hFOB1.19), followed by evaluation of the hFOB1.19 cell proliferation, alkaline phosphatase (ALP) activity and mineralized nodules. The obtained findings indicated that BMSC-Exos promoted the expression of SATB2 in osteoblasts, and SATB2 silencing reduced the ALP activity of osteoblasts and mineralized nodules. MALAT1 acted as a sponge of miR-34c to promote the expression of SATB2. Additionally, BMSCs-derived exosomal MALAT1 promoted osteoblast activity. Moreover, *in vivo* experiments indicated that miR-34c reversed the effect of MALAT1, and SATB2 reversed the effect of miR-34c in ovariectomized mice. Taken together, this study demonstrates that BMSCs-derived exosomal MALAT1 enhances osteoblast activity in osteoporotic mice by mediating the miR-34c/SATB2 axis.

## INTRODUCTION

Osteoporosis remains a significant medical and socioeconomic challenge worldwide, characterized by the systemic impairment of bone mass, and microarchitecture, which ultimately enhances the propensity of fragility fractures [1]. Osteoblasts arise from several types of skeletal stem cells, including skeletal, mesenchymal stem cells (MSCs), with osteogenic differentiation potential [2]. MSCs can differentiate into osteoblasts, chondrocytes, marrow stromal cells, fat cells, tendon cells, and myocytes [3]. Bone marrow-derived mesenchymal stem cells (BMSCs) are crucial components in process of new bone formation. From a therapeutic point of view, BMSCs are relatively easy to obtain and have a low risk of tumor after implantation [4]. In the bone marrow stroma, adipocytes share a common precursor, with an imbalance between osteogenesis and adipogenesis of BMSCs capable of resulting in osteoporosis [5]. Therefore, the discovery of pivotal pathways regulating bone resorption and formation has identified novel approaches with unique mechanisms of action [6].

Interestingly, communication between osteoblasts and osteoclasts has been identified to take place through small membrane-enclosed vesicular particles named as exosomes, which is able to fuse with the surrounding cell membranes within circulatory pathways [7]. Recent studies have demonstrated that osteoblast-derived exosomes regulate osteoclast activity, and exosomes play a role in bone microenvironment and bone metabolism [8, 9]. Intriguingly, long non-coding RNAs (lncRNAs) are emerging as novel modulators during osteogenesis in MSCs [10, 11]. Metastasis-associated lung adenocarcinoma transcript 1 (MALAT1), also known as nuclear-enriched transcript 2 (NEAT2), has been identified as a prognostic biomarker for lung cancer metastasis and has been linked with several other types of human tumors [12]. A previous report concluded that the knockdown of MALAT1 reversed growth inhibition induced by nuclear factor kappa B ligand (RANKL) receptor activator in normal osteoblasts (hFOB1.19) [13]. MicroRNAs (miRNAs) have been shown to regulate multiple processes in bones such as osteoblast and osteoclast differentiation, cell fate management, in addition to orchestration bone programming [14]. miR-containing exosomes derived from osteoclasts selectively inhibited osteoblast activity [15]. Meanwhile, the role of miR-34c has been confirmed in bone homeostasis through affecting both osteoblasts and osteoclasts *in vivo* by regulating several targets (special AT-rich sequence-binding protein 2 [SATB2] and Runx2) in osteoblasts [16]. Enhanced SATB2 has been reported to promote osteogenic differentiation of BMSCs from patients with osteonecrosis induced by ethanol [17]. The current study also reveals that BMSCs-derived exosomes may affect the biological properties of human osteoblasts (hFOB1.19) through SATB2. In order to further explore its potential molecular biological mechanism, we identified by bioinformatics analysis that miR-34c bound to MALAT1 and SATB2, respectively. In our previous research, we identified that MALAT1 stimulated the osteoclastic process in osteoblasts (hFOB1.19) by inhibiting miR-22-5p activity, which could repress osteolysis through blocking the VEGF signaling and enhancing RANKL activity [18]. Based on the aforementioned information, we speculate that exosomal MALAT1 may play a significant role in the progression of osteoporosis by regulating the miR-34c/SATB2 axis.

## RESULTS

### BMSCs-derived exosomes promote osteoblast (hFOB1.19) activity

**Figure 1:**
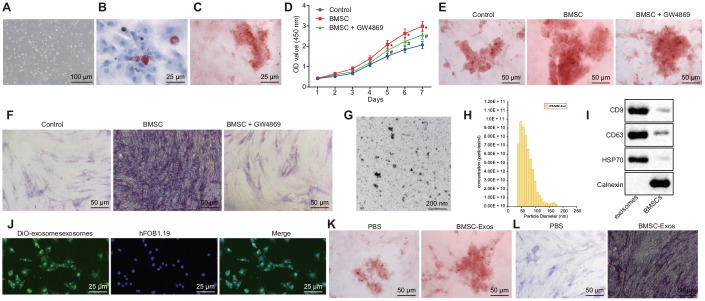
**The exosomes derived from hBMSCs promote the activity of osteoblasts (hFOB1.19).** (**A**) The morphology of BMSCs after 72 h of culture (100 ×). (**B**) Oil red O staining of BMSCs after adipogenic induction for 3 weeks (400 ×). (**C**) Alizarin red staining of BMSCs after osteogenic induction for 3 weeks (400 ×). (**D**) Detection of osteoblasts viability by CCK-8 assay. (**E**) Alizarin red staining of osteoblasts (200 ×). (**F**) ALP staining of osteoblasts (200 ×). (**G**) The morphology of exosome (200 nm) was observed under a TEM. (**H**) Analysis of particle size distribution and concentration in exosome by TRPS. (**I**) The protein expression of CD63, CD9, HSP70 and Calnexin measured by Western blot analysis. (**J**) The endocytosis of hFOB1.19 following 24 h of co-culture with exosomes observed with a confocal microscope (400 ×). (**K**) Alizarin red staining of osteoblasts (hFOB1.19) treated with BMSC-Exos (200 ×). (**L**) ALP staining of osteoblasts (hFOB1.19) treated with BMSC-Exos (200 ×). * *p* < 0.05 *vs.* PBS or control; # *p* < 0.05 *vs.* BMSCs. Data were expressed with mean ± standard error. In Panel **D**, the repeated measures analysis of variance was used for data analysis, followed by Tukey’s post hoc test. The experiment was repeated three times.

The primary cells of hBMSCs were originally mononuclear cells, uneven in size and round shape. The cells began to adhere to the wells 24 h after culture, with spindle and polygonal cells detected 72 h after culture. The morphology of the BMSCs after 72 h of culture is illustrated in Figure 1A. After 3 weeks of adipogenic induction, fat vacuoles in BMSCs were stained in red with oil red O staining considered to be positive (Figure 1B). The osteoblasts were stained with Alizalin red. After 3 weeks of osteogenic induction, the calcium deposit in the BMSCs was stained in red with alizarin red staining considered to be positive (Figure 1C). The aforementioned results demonstrated that the isolated BMSCs could differentiate into adipocytes in addition to revealing that osteoblasts have the ability of multisystem differentiation.

Next, BMSCs were co-cultured with human osteoblasts (hFOB1.19) using the Transwell system. The results indicated that co-culture of BMSCs and osteoblasts (hFOB1.19) could promote the proliferation of human osteoblasts (hFOB1.19) and enhance the calcified nodules as well as ALP activity. GW4869 is a vesicular secretion inhibitor, which is often applied to inhibit the secretion of exosomes. Our results demonstrated that after the addition of GW4869, the degree of promotion on human osteoblasts (hFOB1.19) by BMSCs was diminished (Figure 1D–1F). The aforementioned results demonstrated the effects of co-culture of BMSCs and osteoblasts (hFOB1.19) on the proliferation of osteoblasts (hFOB1.19) and the calcified nodules and ALP activity may be mediated by exosomes. Next, we collected and isolated exosomes from hBMSCs. transmission electron microscopy (TEM) and Tunable resistive pulse sensing (TRPS) analysis revealed that the exosomes were cup-shaped or circular, and the diameter of exosome was 50–150 nm (81.7 ± 17.9) (Figure 1G–1H). Western blot analysis provided evidence indicating that the BMSC-Exos exhibited expression of the exosome markers CD63, CD9 and HSP70 without the expression of Calnexi (Figure 1I), suggesting that we had indeed isolated exosomes. In order to evaluate the ability of BMSCs-derived exosomes to enter human osteoblast hFOB1.19, the exosomes labeled by Dio (green) were co-cultured with human osteoblasts (hFOB1.19) at 37°C for 24 h and then analyzed under a confocal microscope. Our findings revealed that the exosome was endocytosed by osteoblasts (hFOB1.19) (Figure 1J). In order to further verify the effect of co-culture on the proliferation of osteoblasts (hFOB1.19), the calcified nodules as well as the ALP activity was mediated by the exosomes. The isolated BMSCs-derived exosomes were used to treat human osteoblasts (hFOB1.19). The results of alizarin red and ALP staining illustrated that there were more calcified nodules and higher ALP staining intensity in osteoblasts (hFOB1.19) treated with BMSC-Exos than that treated with PBS (Figure 1K–1L). Collectively, the results suggest that hBMSCs promote the activity of osteoblasts (hFOB1.19) through the mediation of exosomes.

### BMSC-Exos promotes SATB2 protein expression in osteoblasts (hFOB1.19)

**Figure 2:**
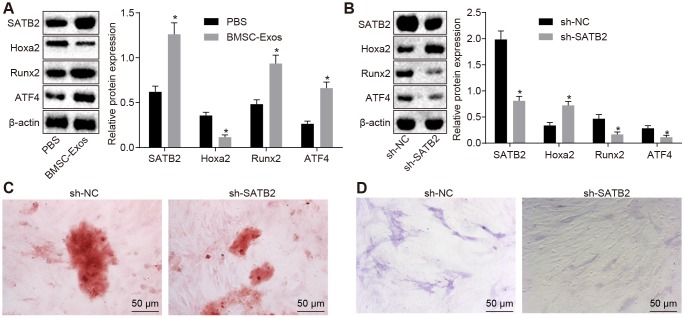
**BMSC-Exos promote the protein expression of SATB2 in osteoblasts (hFOB1.19).** (**A**) The protein expression of SATB2, Runx2, ATF4 and Hoxa2 in osteoblasts (hFOB1.19) treated with BMSC-Exos detected by Western blot analysis. (**B**) The protein expression of SATB2, Runx2, ATF4 and Hoxa2 in osteoblasts (hFOB1.19) treated with sh-SATB2 detected by Western blot analysis. (**C**) Alizarin red staining of osteoblasts (hFOB1.19) treated with sh-SATB2 (200 ×). (**D**) ALP staining of osteoblasts (hFOB1.19) treated with sh-SATB2 (200 ×). * *p* < 0.05 *vs.* sh-NC. Data were expressed with mean ± standard error. In Panel **A** and **B**, the unpaired *t* test was used for data analysis. The experiment was repeated three times.

Previous reports have indicated that the expression of SATB2 in osteoblasts acts to aid in the regulation of osteoblast differentiation [19]. In order to investigate whether the effect of BMSC-Exos on osteoblasts was completed by altering SATB2, Western blot analysis was conducted on osteoblasts (hFOB1.19). Compared with osteoblasts (hFOB1.19) treated with PBS, the protein expression of SATB2, Runx2 and ATF4 was significantly increased while the Hoxa2 protein expression was markedly decreased in the osteoblasts (hFOB1.19) treated with BMSC-Exos (Figure 2A). Next, we set out to further explore the role of SATB2 in osteoblasts, hFOB1.19 was then treated with SATB2 knockdown. The results demonstrated that the protein expression of SATB2, Runx2 and ATF4 was notably reduced, while the protein expression of Hoxa2 was considerably elevated in the osteoblasts (hFOB1.19) treated with si-SATB2. In addition, the results of alizarin red staining and ALP staining indicated that calcified nodules were decreased, in addition to a decrease in the ALP staining intensity in the osteoblasts (hFOB1.19) treated with sh-SATB2 (Figure 2B–2D), all of which suggested that silencing SATB2 could inhibit the osteogenic ability of osteoblasts.

### MALAT1 promotes the expression of SATB2 by binding to miR-34c

**Figure 3:**
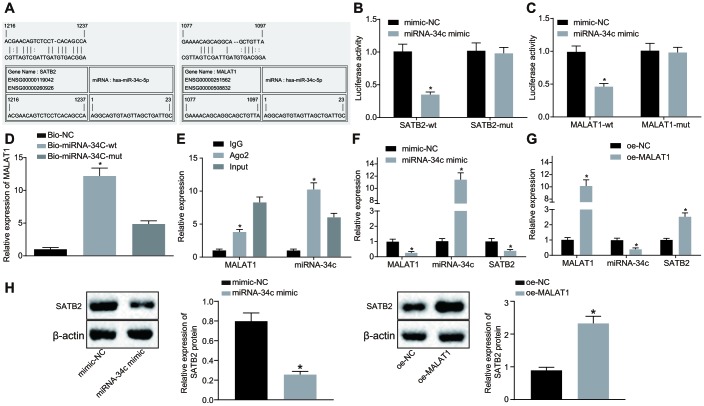
**MALAT1 acts as a sponge of miR-34c to upregulate the expression of SATB2.** (**A**) Prediction of binding sites between miR-34c and MALAT1 as well as those between miR-34c and SATB2 by RNA22. (**B**) The binding between miR-34c and SATB2 as assessed by luciferase activity assay. (**C**) The binding between miR-34c and MALAT1 as assessed by luciferase activity assay. (**D**) The binding of miR-34c and MALAT1 as assessed by RNA pull-down assay, * *p* < 0.05 *vs.* Bio-miR-34c-mut. (**E**) The binding of miR-34c and MALAT1 as assessed by RIP assay, * *p* < 0.05 *vs.* IgG. (**F**) The MALAT1 and miR-34c expression as well as the mRNA expression of SATB2 in osteoblasts (hFOB1.19) treated with miR-34c mimic detected by RT-qPCR. (**G**) The MALAT1 and miR-34c expression as well as the mRNA expression of SATB2 in osteoblasts (hFOB1.19) treated with oe-MALAT1 detected by RT-qPCR, * *p* < 0.05 *vs.* mimic-NC or oe-NC. (**H**) the protein expression of SATB2 in osteoblasts (hFOB1.19) treated with oe-MALAT1 or miR-34c mimic measured by Western blot analysis. Data were expressed with mean ± standard error. In Panel **D** and **E**, the one-way analysis of variance was used for data analysis, followed by Tukey’s post hoc test. in Panel **B**, **C**, **F**, **G** and **H**, the unpaired *t* test was used for data analysis. The experiment was repeated three times.

Based on the above-mentioned results, we identified that BMSC-Exos promoted the osteogenic ability of osteoblasts by the SATB2 gene on osteoblasts. Previous studies have demonstrated that exosomes may potentially exert their effects on biological function through transferring lncRNAs. Therefore, we assumed the position that lncRNAs targets SATB2 gene in BMSC-Exos. Based on the predicted results from bioinformatics website, a putative binding site was identified between miR-34c and MALAT1 or miR-34c and SATB2 3’UTR (Figure 3A). Furthermore, the results of the dual luciferase reporter gene assay demonstrated that the luciferase activity of the vector containing the SATB2 3’UTR-wt was decreased in cells transfected with miR-34c mimic, while the vector containing the SATB2 3’UTR-mut showed no significant change (Figure 3B). Similarly, the luciferase activity of the vector containing the MALAT1-wt was diminished in the cells transfected with miR-34c mimic, while no significant difference was detected in the vector containing the MALAT1-mut (Figure 3C). Therefore, we generate the conclusion that miR-34c could bind to both MALAT1 and SATB2 and played a regulatory role. To further verify this finding, we used the RNA pull-down assay, suggesting that compared with Bio-miR-34c-mut, the MALAT1 bound to Bio-miR-34c-wt was markedly elevated, indicating that miR-34c and MALAT1 could bind to each other (Figure 3D). The Ago2 protein is predominately present in RNA-induced silencing complex (RISC) where miRNAs exert a variety of biological functions. The regulation of miRNA on other RNAs was investigated by RNA-immunoprecipitation (RIP) on the Ago2 protein. We identified that Ago2-bound MALAT1 and miR-34c were significantly increased, confirming the interaction between miR-34c and MALAT1 (Figure 3E). In addition, our study further revealed that osteoblasts (hFOB1.19) treated with miR-34c mimic led to a reduction in the expression of MALAT1 and SATB2 along with an increased expression of miR-34c (Figure 3F, 3H). Furthermore, our results revealed that osteoblasts (hFOB1.19) treated with oe-MALAT1 led to an increase in the expression of MALAT1 and SATB2 and a decline in the expression of miR-34c (Figure 3G, 3H). Our results suggested that MALAT1 can bind to miR-34c and promote the expression of SATB2.

### BMSCs-derived exosomal MALAT1 promotes osteoblast activity

**Figure 4:**
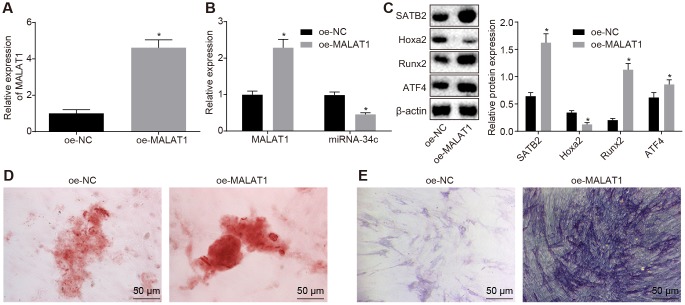
**BMSCs-derived exosomal MALAT1 promotes osteoblast activity.** (**A**) The MALAT1 expression in exosomes evaluated by RT-qPCR. (**B**) The MALAT1 and miR-34c expression in hFOB1.19 detected by RT-qPCR. (**C**) The protein expression of SATB2, Runx2, ATF4 and Hoxa2 in osteoblasts (hFOB1.19) treated with oe-MALAT1 detected by Western blot analysis. (**C**) Alizarin red staining of osteoblasts (hFOB1.19) treated with oe-MALAT1 (200 ×). L. ALP staining of osteoblasts (hFOB1.19) treated with oe-MALAT1 (200 ×). * *p* < 0.05 *vs.* oe-NC. Data were expressed with mean ± standard error. In Panel **A**–**C**, the unpaired *t* test was used for data analysis. The experiment was repeated three times.

Based on the aforementioned findings, we postulated that the BMSCs-derived exosomal MALAT1 could be transferred to osteoblasts (hFOB1.19), while MALAT1 enhances the SATB2 expression in osteoblasts by sponging miR-34c, thus regulating the biological functions of SATB2 related to osteogenic function. Next, the exosomes with overexpressed MALAT1 in BMSCs were collected. Based on the RT-qPCR results, we detected that MALAT1 was significantly enriched in the exosome in the BMSCs with overexpressed MALAT1 (Figure 4A). These exosomes were co-cultured with osteoblasts (hFOB1.19), with the MALAT1 expression in the osteoblasts (hFOB1.19) detected by RT-qPCR found to be consistent with that in Figure 4A. Furthermore, we found that the expression of miR-34c in hFOB1.19 was reduced (Figure 4B). The Western blot analysis results indicated that osteoblasts (hFOB1.19) treated with oe-MALAT1 exhibited increased expression of SATB2, Runx2 and ATF4 as well as decreased expression of Hoxa2 (Figure 4C). The results of both the alizarin red staining and ALP staining revealed there was elevated calcified nodules and ALP staining intensity in the osteoblasts (hFOB1.19) treated with exosomal MALAT overexpression (Figure 4D–4E). These results suggest that BMSCs-derived exosomal MALAT1 promotes osteoblast activity.

### Upregulated MALAT1 alleviates the symptoms of osteoporosis in mice

**Figure 5:**
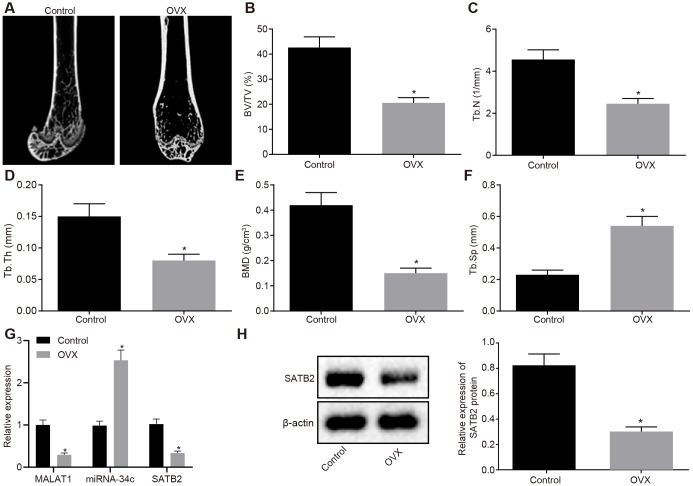
**Characterization of the OVX mouse model.** (**A**) Three-dimensional CT image of distal femur (1 mm). (**B**–**F**) Quantitative data of BV/TV (**B**), Tb.N (**C**), Tb.Th (**D**), BMD (**E**) and Tb.Sp (**F**) in distal femoral of mice. (**G**) The MALAT1 and miR-34c expression as well as the mRNA expression of SATB2 in femoral tissues evaluated by RT-qPCR. (**H**) The protein expression of SATB2 in femoral tissues evaluated by Western blot analysis. * *p* < 0.05 *vs.* control mice. Data were expressed with mean ± standard deviation. In Panel **B**–**F**, the unpaired *t* test was used for data analysis.

In order to confirm successful modeling, we initially conducted Micro-CT 3D imaging analysis of the distal femur of control mice and ovariectomized (OVX) mice. The results indicated that compared with control mice, OVX mice had considerably thinner bone trabeculae, disordered structure of bone trabecula, lower intertrabecular connectivity, increased cortical bone separation and thinner bone cortex (Figure 5A). The quantitative results of the microstructure parameters demonstrated that the bone volume fraction (BV/TV), the bone trabecula number (Tb. N), the bone trabecula thickness (Tb. Th) and the bone mineral density (BMD) values of the OVX mice were significantly decreased when compared with the control mice, with the value of the bone trabecula separation (Tb. Sp) found to be significantly enhanced (Figure 5B–5F). The results demonstrated that the OVX mice were significantly different from the control mice, and the model of the osteoporotic mice was established. The expression of MALAT1, miR-34c and SATB2 in OVX and control mice was measured by RT-qPCR and Western blot analysis, respectively. Our results provided confirmation illustrating that when compared to the control mice, the expression of MALAT1 and mRNA and protein expression of SATB2 were significantly decreased, while the expression of miR-34 was increased (Figure 5G–5H).

**Figure 6:**
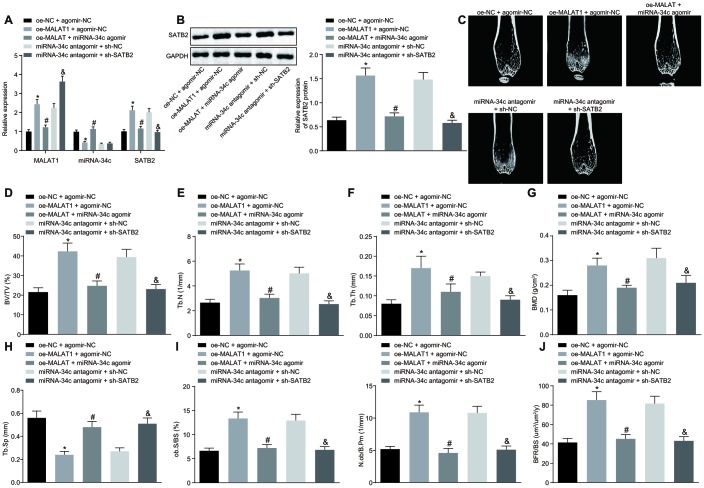
**Upregulated MALAT1 alleviates the symptoms of osteoporosis in mice. OVX mice were injected with oe-MALAT1 + miR-34c agomir or miR-34c antagomir + sh-SATB2.** (**A**) The MALAT1 and miR-34c expression as well as the mRNA expression of SATB2 in femoral tissue was detected by RT-qPCR. (**B**) The protein expression of SATB2 in femoral tissue by Western blot analysis. (**C**) Three-dimensional CT image of distal femur (1 mm). (**D**–**H**) Quantitative data of BV/TV (**D**), Tb.N (**E**), Tb.Th (**F**), BMD (**G**) and Tb.Sp (**H**) in distal femoral of mice. (**I**) Number of osteoblasts in vertebral body sections. (**J**) Calcitonin double labeling analysis. * *p* < 0.05 *vs.* oe-NC + agomir-NC; # *p* < 0.05 *vs.* oe-MALAT1 + agomir-NC; & *p* < 0.05 *vs.* miR-34c antagomir + sh-NC. Data were expressed with mean ± standard deviation. In Panel **B**–**F**, the unpaired *t* test was used for data analysis.

Next, we injected oe-MALAT1, miR-34c agomir, miR-34c antagomir or sh-SATB2 into successfully modeled OVX mice. The expression of MALAT1, miR-34c and SATB2 in mouse femoral tissues was detected by RT-qPCR, the results of which indicated that mice injected with oe-MALAT1 + agomir-NC exhibited elevated expression of MALAT1 and SATB2 while decreased expression of miR-34c, and the mice injected with oe-MALAT1 + miR-34c agomir exhibited a reduced expression of MALAT1 and SATB2 while elevated miR-34c expression was identified. Elevated expression of MALAT1 and reduced expression of SATB2 were detected in the mice injected with miR-34c antagomir plus sh-SATB2 compared to mice injected with miR-34c antagomir and sh-NC (Figure 6A). The protein expression of SATB2 detected by Western blot analysis was consistent with the mRNA expression of SATB2 (Figure 6B). Next, the micro-CT 3D image of the distal femur of mouse revealed signs of a thickened bone trabecula, increased intertrabecular connectivity, decreased cortical bone separation, and thickened cortical bone were found in mice injected with oe-MALAT1. However, in the mice injected with miR-34c agomir or sh-SATB2, our results revealed signs of a thinned bone trabecula, disordered structure of bone trabecula, decreased intertrabecular connectivity, increased cortical bone separation, and thinned cortical bone (Figure 6C). Quantitative analysis of the microstructure parameters indicated that mice injected with oe-MALAT1 exhibited increased values of BV/TV, Tb.N, Tb.Th and BMD in distal femur, while the value of Tb. Sp decreased significantly. Meanwhile, in mice injected with miR-34c agomir or sh-SATB2, decreased values of BV/TV, Tb.N, Tb.Th and BMD in distal femur as well as increased value of Tb. Sp were detected (Figure 6D–6H). Bone histomorphometry analysis of mouse vertebra exhibited an increase in the number of osteoblasts in mice injected with oe-MALAT1, while decreased levels were identified in mice injected with miR-34c agomir or sh-SATB2 (Figure 6I). Based on the analysis of calcitonin double labeling, increased bone formation rate was detected in mice injected with oe-MALAT1, while the bone formation rate in the mice injected with miR-34c agomir or sh-SATB2 was decreased (Figure 6J). Taken together, the results suggest that oe-MALAT1, oe-SATB2 or si-miRNA-34c could help prevent osteoporosis in OVX mice.

## DISCUSSION

In recent years, promising therapeutic approaches to treat osteoporosis are mainly focused on targeting the functions of skeletal stem cells and osteoblasts, which has been proved to be effective in promoting bone formation [2]. Hence, a more detailed understanding of bone biology has led to the identification of novel therapeutic targets with enhanced molecular insights into the communication between bone-forming osteoblasts and bone-resorbing osteoclasts as well as the orchestrating signaling network [1]. Thus, it is necessary to develop new approaches to stimulate osteoblast activity. In the present study, we demonstrate that BMSC-Exos carrying MALAT1 could effectively stimulate the osteoblast activity. Our results highlighted the potential of exosomal MALAT1 to prevent osteoporosis in OVX mouse models.

A key finding of the current study indicated that BMSCs-derived exosomal MALAT1 could potentially promote osteoblast activity. Exosomes could actively transport and transfer information between miRNAs, proteins and mRNAs to target cells, thus affecting their behaviors and strongly modifying the entire microenvironment [20]. LncRNAs including MALAT1 have been found to be overexpressed in exosomes derived from HeLa and MCF-7 cells [21]. Human umbilical cord mesenchymal stem cell-derived exosomes bearing MALAT1 could prevent aging-induced cardiac dysfunction [22]. A previous study demonstrated that human adipose-derived stem cells (hASCs)-derived exosomes containing MALAT1 had a positive effect on function and pathology following a controlled cortical impact (CCI) injury [23]. Consistent with previous reports, we observed the protective role of exosome-mediated delivery of MALAT1 in disease. Furthermore, we detected that the upregulation of MALAT1 could attenuate the symptoms of osteoporosis in mice. Existing literature has suggested that lncRNAs play critical roles in the initiation and pathogenesis of osteoporosis. For instance, a recent study demonstrated that lncRNA MEG3 suppressed the osteogenic differentiation of MSCs in postmenopausal osteoporosis [24]. Tong et al. implicated lncRNA DANCR in the pathology of osteoporosis, highlighting its potential as a biomarker for postmenopausal osteoporosis [25]. Consistent with the findings of the current study, Yang et al. demonstrated the suppressive effect of downregulated MALAT1 on the osteoclastic process in cells, and they verified that MALAT1 influenced the development of osteolysis through the osteoprotegerin (OPG)/RANKL pathway [18].

In addition, our results demonstrated that BMSC-Exos were capable of promoting the expression of SATB2 protein in osteoblasts (hFOB1.19) by binding to miR-34c. A recent study indicated that MALAT1 induces the osteoblast differentiation in human aortic valve interstitial cells by sponging miR-204 [26]. Additionally, it has been reported that MALAT1 regulates the osteogenic differentiation of hBMSCs by directly targeting miR-143 [27]. Preliminary evidence suggests that specific miRNAs regulate osteogenic differentiation in part by modulating master transcription factors and signaling pathways associated with the respective lineages [28]. A large number of the differentially expressed miRNAs have been found to be involved in the early stage of SATB2-induced osteogenic differentiation by participating in the TGF-b/bone morphogenetic protein (BMP) signaling pathway [28]. SATB2 is a specific immunohistochemical biomarker of osteoblastic differentiation and has been shown to be useful for both bone and soft tissue tumors [29]. Genetic evidence in a mouse experiment revealed that SATB2 is a target gene of miR-34b/c in osteoblasts developing during skeletogenesis [30], which was consistent with the findings of the current study. Another study elucidated a regulatory loop in osteoblastic differentiation, including Runx2, SATB2, and miR-31 [31]. SATB2 acts as a protein scaffold to enhance the activity of two transcription factors, Runx2 and ATF4, which play vital roles in promoting osteogenic differentiation [19]. Moreover, evidence has been presented indicating that SATB2 is closely correlated with the function of BMSCs, osteogenic activity and osteoporosis [32–34].

**Figure 7:**
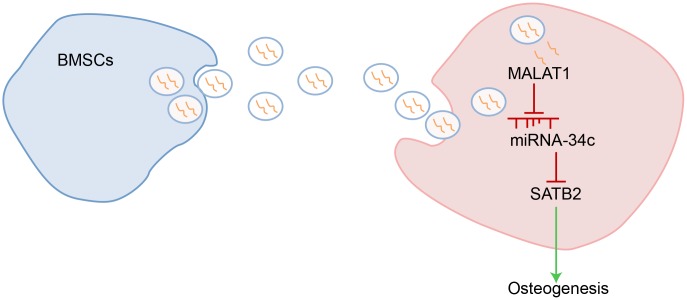
**Schematic representation of the regulatory role of BMSCs-derived exosomal MALAT1 in osteoporosis by mediating miR-34c/SATB2 axis.** BMSCs-derived exosomal MALAT1 acts as a sponge of miR-34c to increase the expression of SATB2, which is conducive to promoting osteogenesis in osteoporosis.

In summary, our study provides evidence that BMSCs-derived exosomal MALAT1 may contribute to enhanced osteogenic activity and alleviated symptoms of osteoporosis in the mouse model by acting as a miR-34c sponge to upregulate SATB2 expression (Figure 7). These results provide a broader understanding of the pathogenesis of osteoporosis as well as novel therapeutic strategies for its treatment. However, further experiments in the future are required to characterize whether miR-34c and MALAT1 might form a competing endogenous RNA network, and to validate the expression pattern of MALAT1, miR-34c and SATB2 in clinical samples. In addition, the effect of MALAT1 on osteogenic differentiation may be explored in transgenic mice in near future.

## MATERIALS AND METHODS

### Ethics statement

The study was conducted under the approval of the Ethics Committee of Xiangya Hospital, Central South University. The informed written consent was obtained from each participant. All animal care, handling, and surgical techniques followed protocols approved by the Animal Care and Use Committee of Xiangya Hospital, Central South University.

### Cell culture

Femoral bone marrow donated by trauma patients was collected under sterile conditions and transferred to a tube containing heparin anticoagulant (with the informed consent of the patient). The bone marrow blood was then placed into a 15 mL centrifuge tube, supplemented with the an identical volume of phosphate buffer saline (PBS) and centrifuged at 403 × g for 20 min in order to discard the fat layer. Ficoll separation solution (5 mL; 077 g/mL) was added to a new centrifuge tube. The diluted bone marrow fluid was added to the upper layer of the Ficoll separation solution along the wall of the tube, and then centrifuged at 1118 × g for 20 min at room temperature. The white cell layer from the centrifuge tube was subsequently transferred to another centrifuge tube, resuspended with PBS and centrifuged at 1509 × g for 15 min. The procedure was repeated twice. The cells were cultured on Dulbecco’s modified Eagle’s medium (DMEM)/F-12 complete culture medium (Invitrogen, Carlsbad, CA, USA) containing 10% fetal bovine serum (FBS) for 72 h. Following the removal of the culture medium and non-adherent cells, the cells were then maintained in DMEM/F-12 complete medium for cell passage.

BMSCs at passage 4-6 were adjusted with the cell concentration. The cells were cultured in a 6-well plate for 24 h at a density of 2 × 10^4^ cells per well. After adherence to the wells, the cells were induced with the adipose differentiation induction medium for 3 weeks. The oil red O staining was applied in order to detect intracellular fat staining. In addition, the culture medium was also renewed with osteoblast differentiation induction medium after cell adherence. After induction for 3 weeks, the cells were stained with alizarin red and analyzed under a microscope.

Human osteoblasts (hFOB1.19) purchased from American Type Culture Collection (ATCC, Manassas, VA, USA) were cultured with DMEM/F12 (Invitrogen, Carlsbad, CA, USA) containing 10% FBS (GE Healthcare Life Sciences, Hyclone, UT, USA) and 1% penicillin and streptomycin (Gibco, Invitrogen, NY, USA) [35].

### Extraction of exosomes from human BMSCs (hBMSCs)

When the BMSC confluence reached 80-90%, the culture medium was renewed with MGro-500 chemically-defined serum-free MSC medium (StemRD) for 48 h. The supernatant was then collected and centrifuged at 300 ×g for 10 min and at 2000 ×g for 10 min in order to ensure removal of the dead cells and cellular debris, respectively. The supernatant was filtered by Steritop^TM^ 0.22 μm aseptic membrane (Millipore, Billerica, MA, USA) and centrifuged at 100000 ×g for 2 h. The precipitation was resuspended at 15 mL and centrifuged in a centrifuge filtration device at 4000 ×g until the final volume had been reduced to approximately 200 μL [36]. All procedures were performed at 4°C. The isolated exosomes were immediately used or stored at −80°C for subsequent analyses.

### Identification of exosomes

BMSC-Exos suspension was diluted with PBS, fixed with 2% paraformaldehyde for 30 min at room temperature, and subsequently suspended in PBS and fixed with 2% paraformaldehyde for 30 min at room temperature. An 8 μL sample was then added dropwise onto the EM grids pretreated with an ultraviolet (UV) lamp. After drying for 30 min, the exosome was stained twice (6 min each time) with 1% uranyl acid. Finally, TEM was conducted using a H-7650 type (Hitachi corp., Tokyo, Japan) at 120 kV [15].

TRPS analyses were performed in order to identify BMSC-Exos. TRPS measurements were conducted using a qNano platform using an NP100-rated nanopore (Izon Science, Oxford, UK). The membrane was stretched to 45.0 mm. The CPC100 particles (Izon Science, Oxford, UK) were applied in order to calibrate the size and concentration in accordance with the manufacturer’s instructions. The samples were diluted 1000 times, and the measurement was performed over a 3 min period [36].

The presence of exosomal characteristic surface marker proteins including CD63, CD9 and HSP70 as well as non-exosome marker protein Calnexin were analyzed by Western blot analysis.

### Western blot analysis

The exosomes as well as the cells were lysed with radioimmunoprecipitation assay (RIPA, R0010, Solarbio, Beijing, China), after which they were centrifuged at 10000 ×g for 20 min. The total protein concentration was determined by bicinchoninic acid (BCA) kit (Pierce, Inc., Rockford, IL, USA). The proteins were separated by 10% sodium dodecyl sulfate polyacrylamide gel electrophoresis (SDS-PAGE) and subsequently transferred onto polyvinylidene difluoride (PVDF) membranes and blocked with 5% skim milk powder for 1 h at room temperature. The PVDF membranes were then probed with the primary antibody, rabbit antibodies to CD63 (ab134045, 1:1000), CD9 (ab92726, 1:2000), HSP70 (ab31010, 1:1000), Calnexin (ab22595, 1:1000), SATB2 (ab92446, 1:1000), Hoxa2 (ab85848, 1:1000), Runx2 (ab23981, 1:1000), ATF4 (ab23760, 1:1000) and β-actin (ab8227, 1:1000) overnight. The membranes were then washed three times with Tris-buffered saline Tween 20 (TBST) and incubated with the secondary antibody, goat anti-rabbit antibody to IgG (ab6721, 1:2000) labeled with horseradish peroxidase (HRP) for 1 h. All aforementioned antibodies were purchased from Abcam Inc. (Cambridge, UK). An identical volume of solution A and solution B of enhanced chemiluminescence (ECL) fluorescent detection kit (BB-3501, Amersham Pharmacia, Piscataway, NJ, USA) mixed in a darkroom, and then added dropwise onto the film and exposed in the gel imager. Images were acquired using the Bio-Rad image analysis system (Bio-Rad, Hercules, CA, USA) and analyzed using Quantity One v4.6.2 software. The relative protein content was expressed as the gray value of the corresponding protein band/β-actin protein band. The experiment was repeated three times.

### Cell treatment

Human osteoblasts (hFOB1.19) were transduced with shRNA (sh)-negative control (NC; 1000 ng), sh-SATB2 (1000 ng), mimic-NC (100 nM), miR-34c mimic (100 nM), overexpression vector (oe)-NC (1000 ng) or oe-MALAT1 (1000 ng). BMSCs were transduced with oe-NC or oe-MALAT1. Lentivirus-mediated shRNA targeting SATB2 and MALAT1 overexpression vector were purchased from System Biosciences (SBI, Mountain View, CA, USA). The cells were seeded into a 12-well plate with DMEM medium 24 h prior to treatment. When cell confluence had reached approximately 70%, the cells were resuspended with serum-free DMEM and seeded into the 12-well plates. Human osteoblasts (hFOB1.19)/hBMSCs were infected with 10 μL lentivirus (10^7^ U/mL). Human osteoblasts (hFOB1.19) were transduced with 100 nM miR-34c mimic or mimic-NC by Lipofectamine 2000 as per the manufacturer’s instructions (Invitrogen, Carlsbad, CA, USA). After treatment, the cells were cultured with 5% CO_2_ at 37°C. After 6 h, the culture medium was renewed with a complete culture medium prior to further incubation for 24-48 h. The successfully transduced hBMSCs were employed for exosome isolation.

### Fluorescent labeling and transfer of exosomes

The isolated exosomes were labeled with 10 μM lipophilic staining solution Dio (Thermo Fisher Scientific Inc., Waltham, MA, USA) and incubated for 20 min at 37°C. The labeled exosomes were centrifuged at 12000 g for 90 min at 4°C. The osteoblasts (hFOB1.19) (3 × 10^4^ cells per well) were incubated with 25 μg/mL Dio-labeled exosomes for 24 h in a 24-well plate. The cells were then resuspended in 500 μL of PBS and stained with 4′,6-Diamidino-2-Phenylindole. Finally, the internalization of the exosomes was determined using a confocal microscope.

### Co-culture of osteoblasts and BMSCs

The human osteoblasts (hFOB1.19) were seeded into a 6-well plate at a density of 2 × 10^4^ cells per well, and cultured with 2 mL DMEM in an incubator with CO_2_ for 24 h in order to ensure complete adherence to the well. The human osteoblasts (hFOB1.19) were treated with exosome-free medium, cultured with BMSCs by transwell system, cultured with BMSCs and supplemented with exosome blocker (GW4869, 10 μM, Sigma-Aldrich, St. Louis, MO, USA), 50 μL BMSC-Exos suspension, 50 μL PBS, 50 μL BMSC-Exos suspension of NC, or 50 μL BMSC-Exos suspension of MALAT1 overexpression, respectively.

### Alkaline phosphatase (ALP) staining and alizarin red staining

Human osteoblasts (hFOB1.19) were cultured for 14 days in fresh osteogenic differentiation medium containing 50 M ascorbic acid-2-phosphate, 10 mM β-glycerophosphate and 100 nM dexamethasone. ALP staining was then performed in accordance with the instructions of the kit (Beyotime Institute of Biotechnology, Shanghai, China), with the staining outcome analyzed under a microscope. Bone mineralization was detected by alizarin red staining as per the instructions of the kit (Sigma-Aldrich, St Louis, MO, USA). Human osteoblasts (hFOB1.19) were stained with 2% alizarin red (pH = 4.2) for 10 min, and then washed with distilled water. The mineralized nodules were examined with a phase contrast microscopy on the 21^st^ day [36, 37].

### Cell counting kit-8 (CCK-8) assay

The osteoblasts were seeded into 96-well plates at a density of 2 × 10^3^ cells per well. The treated cells were then cultured for 24 h in an incubator with CO_2_ for complete adherence to the well. After 24 h, the cells in each well were incubated with 10 μL CCK-8 solution (Dojindo Co. Tokyo, Japan) at 37°C for 2.5 h. The incubated plate was then placed into a microplate reader in order to determine the optical density (OD) value at the wavelength of 450 nm. The culture medium in each well post color development was subsequently renewed with fresh culture medium. The OD value was measured every 24 h for 7 d. A curve was plotted based on the OD value. The experiment was repeated three times in each group.

### Dual luciferase reporter gene assay

The synthetic SATB2 3’untranslated region (UTR) gene fragment was introduced into pMIR-reporter (Beijing Huayuyang Biotechnology Co., Ltd., Beijing, China) by endonuclease Spe I and Hind III in order to design complementary mutation sites of seed sequence on SATB2 wild type (wt). After restriction endonuclease digestion, the target fragment was inserted into the pMIR-reporter plasmid with T4 DNA ligase. The correctly sequenced luciferase reporter plasmids wt and mutant type (mut) were co-transfected with miR-34c into HEK-293T cells (CRL-1415, Shanghai Xinyu Biotechnology Co., Ltd., Shanghai, China). After 48 h of transfection, the cells were harvested and lysed. A luciferase assay kit (RG005, Shanghai Beyotime Biotechnology Co., Ltd., Shanghai, China) as well as a Glomax20/20 luminometer fluorescence detector (Promega, Madison, WI, USA) was employed to detect luciferase activity. The relationship between MALAT1 and miR-34c was detected using the same method. The experiment was repeated three times in each group.

### RNA pull-down assay

The osteoblasts (hFOB1.19) were treated with 50 nM biotin-labeled Bio-miR-34c-wt, Bio-miR-34c-mut and the corresponding Bio-NC for 48 h. The cells were incubated in specific lysis buffer (Ambion, Austin, TX, USA) for 10 min, and then centrifuged at 14000 ×g in order to extract the supernatant. The protein lysate was incubated with M-280 streptavidin magnetic beads (S3762, Sigma-Aldrich, St Louis, MO, USA) that had been pre-coated with bovine serum albumin (BSA) without RNase and yeast tRNA (TRNABAK-RO, Sigma-Aldrich, St Louis, MO, USA). The beads were incubated at 4°C for 3 h, washed twice with pre-cooled pyrolysis buffer, three times with low salt buffer and once with high salt buffer. The bound RNA was purified by Trizol, and then MALAT1 expression was detected by reverse transcription quantitative polymerase chain reaction (RT-qPCR).

### RNA-immunoprecipitation (RIP)

The osteoblasts (hFOB1.19) were lysed with lysis buffer containing 25 mM Tris-HCl (pH = 7.4), 150 mM NaCl, 0.5% NP-40, 2 mM ethylene diamine tetraacetic acid, 1 mM NaF and 0.5 mM dithiothreitol supplemented with RNasin (Takara Bio Inc., Shiga, Japan) and protease inhibitor mixture (B14001a, Roche, Indianapolis, IN, USA). The lysate was centrifuged at 12000 ×g for 30 min, then incubated with anti-Ago2 magnetic beads (130-061-101, Shanghai Univ-bio, Shanghai, China) at 4°C for 4 h with anti-IgG magnetic beads as NC. The beads were washed three times with wash buffer containing 50 mM Tris-HCl, 300 mM NaCl (pH = 7.4), 1 mM MgCl_2_ and 0.1 NP-40. The RNA was extracted from the magnetic beads by Trizol, and the expression of MALAT1 and miR-34c in the osteoblasts was detected by RT-qPCR.

### RT-qPCR

The total RNA was extracted from the exosomes and cells by Trizol (Invitrogen, Carlsbad, CA, USA), and subsequently reversely transcribed into cDNA based in accordance with the cDNA kit manufacturer’s instructions (K1622; Fermentas Inc., Ontario, CA, USA). The expression of miR-34c, MALAT1 and SATB2 was determined by SYBR Premix Ex Taq II Kit (Takara Bio Inc., Shiga, Japan). U6 was regarded as the internal reference for miR-34c while GAPDH was applied as the internal reference for MALAT1 and SATB2. The primer sequences for genes are depicted in Table 1. 2^-ΔΔCt^ illustrated the fold changes in the target gene expression between the experimental group and the control group. The experiment was repeated three times.

**Table 1:** Primer sequence for RT-qPCR.

**Gene**	**Forward primer sequence**	**Reverse primer sequence**
miR-34c	5′-ACACTCCAGCTGGGAGGCAGTGTAGTTAGCT-3′	5′-TGGTGTCGTGGAGTCG-3′
U6	5′-CGCTTCGGCAGCACATATACTAAAATTGGAAC-3′	5′-GCTTCACGAATTTGCGTGTCATCCTTGC-3′
MALAT1	5′-AAAGCAAGGTCTCCCCACAAG-3′	5′-GGTCTGTGCTAGATCAAAAGGCA-3′
GAPDH	5′-GGAGCGAGATCCCTCCAAAAT-3′	5′-GGCTGTTGTCATACTTCTCATGG-3′
SATB2	5′-GCAGTTGGACGGCTCTCTT-3′	5′-CACCTTCCCAGCTTGATTATTCC-3′

### Establishment of an OVX mouse model

All operations were performed under general anesthesia, with postoperative nursing care performed with anti-aminophenyl alcohol in order to minimize animal pain and suffering. Twenty-four adult female BALB/c mice (9 weeks old, average weight of 18-22 g; Hunan Experimental Animal Center, Changsha, Hunan, China) were selected and randomly assigned into two groups: OVX group and control group (n = 12). Mice were anaesthetized with pentobarbital sodium at 30 mg/kg, and two bilateral 10 mm diameter incision was made on lateral lumbar skin. The bilateral ovaries of the mice were cautiously removed by exposing the muscles and retroperitoneum by blunt dissection. All animals in the control group received the same procedure without the removal of bilateral ovaries. The tissue was then repositioned and sutured. The mice were injected with 40000 IU/mL penicillin at 1 mL/kg for 3 d. Two months after OVX model establishment, the distal femur was obtained to confirm the occurrence of osteoporosis using micro-CT analysis [36, 38].

### Treatment of the OVX mouse model

The successfully established mouse models were administered with either oe-MALAT1 (20 μL) or control BMSC-Exos suspension (20 μL), sh-SATB2 (10^7^U/mL) or sh-NC lentivirus a dose of 5 μL, miR-34c agomir/ antagomir (10 nM) or mutant agomir-miR-34c (10 nM, agomir-NC) twice per week by periosteal injection into the marrow cavity of the femur [33].

### Micro-computed tomography (micro-CT)

After 3 weeks, the micro-CT system (mCT-80, Scanco Medical, Brüttisellen, Switzerland) was used to analyze changes of microstructures in osteoporotic models and formation of new bone in the defective area Using the medium resolution mode, the samples were scanned at a thickness of 0.018 mm of each slice, with a 1,024-reconstruction matrix together with a 200 ms integration time. After 3D reconstruction, BMD, BV/TV, Tb.N, Tb.Th and Tb.Sp were automatically determined in order to verify the osteoporotic model, and BMD and BV/TV values in the defect regions were used to evaluate new bone formation by auxiliary software (Scanco Medical, Bassersdorf, Switzerland) [36].

### Skeletal analyses and bone histomorphometry

Toludine blue on 5-7 mm of uncalcified lumber vertebrae plastic sections was performed based on standard protocols. The static and dynamic histomorphometric analysis was performed via calcein injection with 15 mg/kg at 7-day intervals following the first injection, after which data were analyzed on the OsteoMeasure histomorphometry system (Osteometrics, Decatur, GA, USA) [16].

### Statistical analysis

SPSS 21.0 (IBM SPSS Inc. Chicago, IL, USA) statistical software was used to analyze the data. The measurement data were expressed as mean ± standard deviation (error). The unpaired t test was used to compare the data with normal distribution and variance homogeneity between two groups. The one-way analysis of variance (ANOVA) was utilized to analyze the data among multiple groups, and the date at different time points were analyzed by repeated measures ANOVA, followed by Tukey’s post hoc test. *p* < 0.05 indicates the difference was statistically significant.
